# When, and When Not, to Interfere

**Published:** 1898-11

**Authors:** 


### WHEN, AND WHEN NOT, TO INTERFERE.

The normal, that is the usual, position of the uterus causing no discomfort is in the axis of the pelvic inlet, and at an angle to that of the vagina, which may, roughly, be said to lie in the axis of the pelvic outlet. When the uterus is tilted back, it is said to be retroverted, and if bent back, retroflexed. Forward displacements are anteversions and flexions. It is a common custom to replace organs thus dislocated from the common position and hold them thus by means of mechanical appliances, pessaries, whether the malposition is causing any trouble or not. This is a mistake. So it is to stitch up a torn cervix and a lacerated perineum that is causing no trouble. Countless numbers of women have enjoyed perfect comfort despite versions, flexions and lacerations, who were first initiated into the mysteries of chronic genital disturbances after the wanton interference of an officious medical attendant.

"Meddlesome midwifery" is an expressive term born of the home-driven conviction caused by long experience, that the normal course of affairs is often wantonly interfered with to the detriment of the patient. Children's lives are offered up constantly as unnecessary sacrifices to this moloch of useless, nay harmful, interference. Food is changed, clothing is altered, and strong medicines needlessly given, for the mere sake of doing something. The child suffers—the physician ought.

We may safely lay down the rule that when an abnormal condition, in itself not necessarily dangerous or bad, causes no disturbance, it would best be left alone. We have known of a perineal laceration that never caused the slightest discomfort in an active woman during eighteen years, and despite which all the pelvic organs remained in their proper positions. If this is possible in one case, it may be so in others; and what is true of so signal a disturbance of local relations as a torn perineum is more apt to be true of lesser troubles.

Meddlesomeness is the crime of carelessness and ignorance. We should not disturb the harmony of the bodily functions by wanton interference. Too often, the penalty is severe. There is much to lose and nothing immediate to gain. The fact must be ever borne in mind, that in such cases of needless interference, the patient submits to an unknown treatment or operation because of confidence in the judgment and honesty of her adviser. Under the most favorable circumstances, she can feel no better than she did before treatment began, for she felt perfectly well then. Should she feel in any way worse, she is justified in questioning the physician's skill, judgment and honesty.

Many a child's life has been the cost of needless interference during birth; the mother is often injured; and, worst of all, the offender seldom recognizes his error and thus continues his damaging course.

It has often been said that most of the art of medical practice lies in the making of a correct diagnosis, and this is undoubtedly true, for treatment is usually easy enough when a correct and thorough diagnosis has been made. At all events, it enables one to say whether treatment is likely to be of much account or not, or whether really needed or not. And right here is the point we contend for—whether really needed or not. How many men ask themselves this question? Very few. The placebo should be used much oftener than it is. Christian scientists and faith curists, and faddists generally, cure when there is little or no trouble, unless it be such as can be favorably modified by mental suggestion. But a suggestion clothed in some form that may be harmful in itself is bad, should never be used. Operations and potent drugs are bad media for the conveyance of suggestions. This should be distinctly remembered. The tendency to rush from one extreme to the other is so great that fads are the inevitable outcome. It has been so from the most remote times, and is so at the present day, more noticeable now because of the increased diversity of our knowledge, and the consequent greater scope offered for the indulgence of our varied enthusiastic fancies. Common sense, good, staid, unruffable reason, are more needed now in medical practice than ever before. Fads come and go, like the leaves of the trees and the broods of the lesser organisms, but rock-ribbed truth is everlasting. It has a quality that the balanced mind readily detects it by, but which the enthusiast fails to see.

The lesson we would draw from all this is not to repair a past injury that nature has cured in her own way, and which causes no trouble. Also not to wantonly interfere with a patient's food and environment to which she has adapted her self, nor to interfere with the processes of nature when they are normal, or even when abnormal if they are subserving a useful purpose and tending to a desirable end. It is better to use a placebo when in doubt, and suggest to the mind of the patient an abundance of hope, than it is to employ potent remedies, for there is danger that they may be misemployed.

Whilst these are all admittedly trite enough truisms, the other fact remains with distressing clearness, that those truths are not put enough into practice, and that all suffer in consequence—the physician and the general public and the medical profession. Why not come down off our high horses, and first of all do that which we know to be good, then leave undone that which we know may be bad, and finally cheer our patients when we know that we honestly can, and when it will do them good?—*The Medical Council.*

